# Association between Milk and Milk Product Consumption and Anthropometric Measures in Adult Men and Women in India: A Cross-Sectional Study

**DOI:** 10.1371/journal.pone.0060739

**Published:** 2013-04-08

**Authors:** Ambika Satija, Sutapa Agrawal, Liza Bowen, Neha Khandpur, Sanjay Kinra, Dorairaj Prabhakaran, Kolli Srinath Reddy, George Davey Smith, Shah Ebrahim

**Affiliations:** 1 Public Health Foundation of India, New Delhi, India; 2 South Asia Network for Chronic Disease, Public Health Foundation of India, New Delhi, India; 3 Department of Non-communicable Disease Epidemiology, London School of Hygiene and Tropical Medicine, London, United Kingdom; 4 Centre for Chronic Disease Control, New Delhi, India; 5 University of Bristol, Bristol, United Kingdom; Indiana University, United States of America

## Abstract

**Background:**

The nutritional aetiology of obesity remains unclear, especially with regard to the role of dairy products in developing countries.

**Objective:**

To examine whether milk/milk product consumption is associated with obesity and high waist circumference among adult Indians.

**Methods:**

Information on plain milk, tea, curd and buttermilk/*lassi* consumption assessed using a Food Frequency Questionnaire was obtained from the cross-sectional sib-pair designed Indian Migration Study (3698 men and 2659 women), conducted at four factory locations across north, central and south India. The anthropometric measures included were Body Mass Index (BMI) and Waist Circumference (WC). Mixed-effect logistic regression models were conducted to accommodate sib-pair design and adjust for potential confounders.

**Results:**

After controlling for potential confounders, the risk of being obese (BMI≥25 kg/m^2^) was lower among women (OR = 0.57;95%CI:0.43−0.76;p≤0.0001) and men (OR = 0.67;95%CI: 0.51−0.87;p = 0.005), and the risk of a high WC (men: >90 cm; women: >80 cm) was lower among men (OR = 0.71;95%CI:0.54−0.93;p = 0.005) and women (OR = 0.79;95%CI:0.59−1.05;p>0.05) who consume ≥1 portions of plain milk daily than those who do not consume any milk. The inverse association between daily plain milk consumption and obesity was also confirmed in sibling-pair analyses. Daily tea consumption of ≥1 portion was associated with obesity (OR = 1.51;95%CI:1.00−2.25;p>0.050) and high WC (OR = 1.65;95%CI:1.08−2.51;p>0.019) among men but not among women but there was no strong evidence of association of curd and buttermilk/*lassi* consumption with obesity and high waist circumference among both men and women.

**Conclusions:**

The independent, inverse association of daily plain milk consumption with the risk of being obese suggests that high plain milk intake may lower the risk of obesity in adult Indians. However, this is an observational finding and uncontrolled confounding cannot be excluded as an explanation for the association. Therefore, confirmatory studies are needed to clarify this relationship.

## Introduction

India is in a state of transition with marked social, demographic and epidemiological changes underway [1]. This transition is characterised by increased longevity, an increasingly urbanised population, projected to rise from 28% in 2001 to 50% by 2025 [2] and a rising burden of chronic diseases [3]. In 2005 around 53% of India’s mortality was attributed to chronic diseases [4], a figure that is estimated to reach approximately 68% (8 million deaths) by 2020 [3], [4]. This increase has been mirrored by a similar trend – that of escalating levels of obesity [5]. The prevalence of excess body weight has reached epidemic proportions, with more than 1·6 billion adults being overweight (BMI≥25 kg/m^2^) worldwide of which 400 million are clinically obese (BMI≥30 kg/m^2^) [6]. WHO projections suggest that by 2015, approximately 2·3 billion adults will be overweight and more than 700 million obese. Evidence shows that obesity is a risk factor for several chronic diseases and in itself is indicative of changes in activity and dietary patterns of population groups [7].

The change in food intake in Indian populations is characterized by an increase in the consumption of animal protein including dairy products [8], [9]. Published global evidence mostly based on developed or high income countries’ experience on dairy consumption and body weight is extensive but equivocal. Many studies have found a negative association between milk/dairy consumption and body mass index [10]–[16] and central adiposity [17]. However, some studies have found no association [18]–[20], while others have found a positive association [21], [22]. Many studies have also distinguished between low and high fat dairy products, with only the former being associated with a more favourable risk profile [23].

Per capita consumption of dairy products is generally higher in high income countries (HICs) relative to low and middle income countries (LMICs) [24], with average dairy consumption levels of 245 kg/capita/year in HICs compared with only 66.2 kg/capita/year in LMICs [25].

Milk has a long history of usage in India [26]. Cows are worshipped by Hindus and milk from both cows and water buffalo has long been valued as a food [26]. Promotions also emphasize milk’s links to growth, gaining in height and strength [27]. Dairy supply is increasing in India, with a report of annual per capita dairy supply increased from 39 kg to 70 kg during 1980–2003 [28], and according to Food and Agricultural Organisation [25], daily per capita supply of milk increased from 69 kcal in 1980 to 103 kcal in 2007. The National Sample Survey Organisation (NSSO) of India 2004–5 survey found that the proportion of energy from milk products and oils and fats were higher in urban than rural people [29]. The Indian Consumer Expenditure Survey of 2009–2010 found per capita monthly expenditure on milk and milk products was Rs. 81 (US$ 1.5) in rural India and Rs. 137 (US$ 2.55) in urban India [30]. However, despite increases, consumption levels remains low (and far below recommended as well as observed western levels), and some increase in animal products could be beneficial in increasing the amount of protein in the Indian diet [31].

In light of the increasing milk/milk product consumption in India and increasing levels of obesity [32], understanding the role that dairy consumption may be playing in fueling the risk in obesity is important. The objective of this analysis was therefore to assess whether the consumption of milk/milk products was associated with markers of obesity – body mass index (BMI), and waist circumference (WC) among men and women in India.

## Methods

### Study Design

Data from the Indian Migration Study (IMS) was used for this study. The design and sampling methodology of the IMS has been described previously [33]–[35]. Briefly, the IMS is a cross-sectional sib-pair study, part of a larger cardiovascular risk factor surveillance system [36] in industrial populations all over India. The IMS was carried out in factory settings in four cities from northern, central and southern India (Lucknow, Hindustan Aeronautics Ltd; Nagpur, Indorama Synthetics Ltd; Hyderabad, Bharat Heavy Electricals Ltd; and Bangalore, Hindustan Machine Tools Ltd). Information on rural-to-urban migration was solicited from factory workers and their co-resident spouses. Factory workers who had migrated from rural to urban areas, along with a 25% random sample of urban non-migrants, were asked to participate in the study. Each migrant participant was asked to identify a non-migrant sibling residing in a rural area, preferably of the same gender and close to them in age, who was then also invited to participate in the study. In a small number of cases where no rural sibling was available (<5%), a cousin or a close friend (<0.5%) from the same village was invited. There were no other exclusion criteria at this recruitment stage. This sampling strategy resulted in rural dwelling siblings being drawn from anywhere in the country (18 of the 28 Indian states), reflecting the migration patterns of the factory workers and their spouses. A substantial proportion came from the four large states in which the factories were based. The urban participants were also asked to identify a non-migrant, urban dwelling sibling for inclusion in the study. The fieldwork took place between March 2005 and December 2007.

### Anthropometric Measurement

A digital weighing machine with ±100 gm accuracy (Model PS16, Beurer, Germany) was used to weigh the participants, while they were wearing light indoor clothing. For measuring height, the Frankfort plane was used with a portable plastic Stadiometer with a base plate, with 1 mm accuracy (Leicester height measure, Chasmors, London). Two measures of WC were taken at the narrowest part of the abdomen between the ribs and the iliac crest, as seen from the anterior aspect, on bare skin, with a non-stretch metallic tape with a blanket lead-in (Chasmors, London). Obesity was defined as BMI≥ 25 kg/m^2^ following the recommendations of recent Indian consensus groups [37], [38]. A high WC was defined as ≥90 cm in men and ≥80 cm in women following recommendation specific for Indian populations [38], [39].

### Dietary Assessment

Diet was assessed using an interviewer-administered semi-quantitative food frequency questionnaire (FFQ), details of which have been given in a previous publication [40], [41]. Briefly, the questionnaire assessed portion size and frequency of intake of 184 commonly consumed food items, asking about consumption over the last year. A standard portion size was assigned to each food (e.g. teaspoon = 5 ml, tablespoon = 10 ml, ladel = 56 ml, standard glass = 125 ml, medium glass = 143 ml, and bowl = 220 ml), and participants were shown examples of these vessels and asked to report portions consumed as multiples of this. Frequency was recorded as daily, weekly, monthly, yearly/never. A single FFQ was used to cover the four regions of the study.

Out of the 184 food items, the consumption of plain milk (refers to plain milk with or without sugar but not any other additions), consumption of tea, curd (refers to fermented milk) and buttermilk/*lassi* (refers to diluted beaten curds with or without seasonings) were used for the present study as the principal predictor variables. All these milk products exclude those added to beverages and preparations. Consumption of these four items was analysed in terms of numbers of portions consumed per day in grams (g). Amount in one portion: 120 g for plain milk measured in a glass; 124 g for tea measured in a glass; 143 g for buttermilk/*lassi* measured in a glass; 195 g for curd measured in a bowl.

In order to assess the reliability of the IMS-FFQ, sub-samples were asked to complete the questionnaire 1–2 months (n = 185), as well as 12 months (n = 305) after completion of the questionnaire during the original period of data collection. These two sub-samples were randomly selected and were independent of each other. Cohen’s k-statistic was used to compare participants’ tertile grouping across any two FFQ administrations n addition to intra class correlation coefficients calculation. Kappa coefficients for energy intake and dairy products were 0.45 and 0.46 respectively indicating moderate agreement and are similar to values obtained in other reliability studies [42]–[45]. Another 530 participants (53.9% male) were administered a reference method of three 24-hour recalls, which was used to validate the FFQ. Energy intake was over-estimated by FFQ compared with 24 hour recalls by 409 kcals (19%) with a Spearman correlation coefficient of 0.61. Dairy products were also over-estimated by 36 g (18%) with a Spearman correlation coefficient of 0.63. These results are comparable with other Indian FFQ–24 h recall validations, most of which have shown an overestimation of consumption using FFQ relative to recalls and diaries [44], [45].

### Socio-economic, Demographic and Lifestyle Variables

Participants were asked to complete an interviewer-administered questionnaire to gather information on socio-demographic and demographic indicators, including age, socio-economic status, education, occupation, religion, caste/tribe and migration status. The Standard of Living Index (SLI) was calculated by applying standard weights to subsets of questions from a household level asset-based scale devised for Indian surveys, and rescaling them to the full score [34]. The score was then categorised into tertiles to produce low, medium and high socio-economic position (SEP) groups. Measurement at the household level is appropriate in the Indian context, in which the individual’s SEP has less impact on their material wealth. This asset-based score was considered a more appropriate indicator of SEP for these analyses than education, income, or occupation alone, because it is more likely to reflect the changes that migrants experience following their move to urban areas. In the context of developing countries, low SLI is associated with tobacco use [46] and with mortality [47], indicating its validity as a socioeconomic marker.

The questionnaire was also used to ask participants about lifestyle variables, such as current tobacco use in any form (smoked or chewed on a daily basis in the previous six months), and regular consumption of alcohol (on ≥10 days a month in the previous six months). Physical activity was assessed using a quantitative physical activity questionnaire (IMS-PAQ) developed for use in the IMS, adapted and validated for use in Indian populations [48], [49]. This provided information on a participant’s habitual daily activity over the last one month in terms of MET (Metabolic Equivalent of Task) hours/day.

### Quality Assurance of Measurements

All instruments and protocols were piloted before the start of the study. Fieldworkers at the four study sites were trained together and methods were standardised at the outset, and subsequently every six months. The anthropometric equipment was calibrated at the start of every clinic.

### Statistical Analysis

Descriptive analysis was done using chi square tests. The association of milk and milk product consumption with anthropometric indicators was assessed using mixed-effect logistic regression models. Potential confounders controlled for included age, socio-economic status, education, migration, tobacco use, alcohol consumption, energy intake and physical activity.

Analyses were carried out separately in men and women, as we anticipated that there may be gender differences in the effect of milk and milk product intake, and also because of the statistical dependency between husbands and wives produced by the study design. Robust standard errors were used to account for clustering in sibling pairs.

In addition to these standard descriptive analyses we also carried out mixed effects logistic regression analyses proposed for twin studies [50], making use of the sibling-pair design of this study to investigate the extent to which associations may reflect factors shared by the siblings (early home environment and inheritance) as opposed to factors that were different (current environment) [46]. Mixed effects logistic regression models, using population-averaged approach, were fitted for the two primary binary outcomes of BMI and WC incrementally adjusting for confounders analogous to the standard analyses above. We also fitted mixed effects logistic regression models to estimate the association of plain milk, tea, curd, and buttermilk/*lassi* consumption with BMI, and WC separately for within-sibling-pairs and between-sibling-pairs. All analyses were conducted using STATA version 10 (StataCorp, College Station, TX, USA).

### Ethics Approval

Information sheets (translated into local languages) were provided to the participants and informed consent was obtained through their signatures (or a thumb print if the individual was illiterate). Ethics committee approval was obtained from the All India Institute of Medical Sciences Ethics Committee, reference number A-60/4/8/2004, and the procedures followed were in accordance with the ethical standards of the Committee.

## Results

The total IMS sample consisted of 2944 women and 4123 men. For the present study, individuals with known diabetes (n = 486), heart disease (n = 78), stroke (n = 25), and peptic ulcer (n = 121) were excluded as it is possible that diagnosis with these diseases may change the dietary habits of the participants. Thus, the final analysis is based on 3,698 men and 2,659 women aged 18 years and above.

The mean body mass index was 23.0 kg/m^2^ and 24.4 kg/m^2^ for men and women respectively; 30.4% men and 42.9% women were obese. The mean waist circumference was 84.1 (±11.6SD) and 78.1 (±10.8SD) for men and women respectively; 32.8% men and 42.5% women had WC above the threshold for obesity.

### Sample Characteristics

Table 1 shows the distribution of the sample by selected characteristics. Almost half of the participants never consumed plain milk, whereas almost a third consumed <1 portion and almost a quarter consumed ≥1 portion daily. While a majority (>80%) of our sample population drinks ≥1portion of tea daily, a quarter of the participants never consumed buttermilk/*lassi*, 61% consumed <1 portion and 14% consumed ≥1 portion daily. The distribution of curd consumption was similar to that of buttermilk consumption. More than three-fourth of the study participants belonged to the age group 25–54 years. While more than 80% of the participants lived in households with a high standard of living, less than one-tenth lived in households with a low standard of living. Participants were equally distributed across the rural, migrant and urban categories, as well as across the four cities. Between 74–80% of the participants had never consumed tobacco and/or alcohol. Men tended to consume more calories, and be more active than women.

**Table 1:** Sample distribution according to milk/milk product consumption and other selected characteristics, Indian Migration Study, 2006.

Characteristics	Men	Women	Total
	N (%)	N (%)	N (%)
**Daily plain milk consumption** a			
Never	1645 (44.5)	1439 (54.1)	3084 (48.5)
< 1 portion	1095 (29.6)	688 (25.9)	1783 (28.1)
≥ 1 portion	958 (25.9)	532 (20.0)	1490 (23.4)
**Daily tea consumption** a			
Never	254 (6.9)	313 (11.8)	567 (8.9)
< 1 portion	310 (8.4)	262 (9.9)	572 (9.0)
≥ 1 portion	3134 (84.8)	2084 (78.4)	5218 (82.1)
**Daily curd consumption** a			
Never	778 (21.0)	641 (24.1)	1419 (22.3)
< 1 portion	2259 (61.1)	1642 (62.8)	3901 (61.4)
≥ 1 portion	661 (17.9)	376 (14.1)	1037 (16.3)
**Daily buttermilk/** ***lassi*** ** consumption** a			
Never	835 (22.6)	747 (28.1)	1582 (24.9)
< 1 portion	2356 (63.7)	1501 (56.5)	3857 (60.7)
≥ 1 portion	507 (13.7)	411 (15.5)	918 (14.4)
**Age (years)**			
18–25	228 (6.2)	220 (8.3)	448 (7.1)
25–34	981 (26.5)	622 (23.4)	1603 (25.2)
35–44	1033 (27.9)	957 (36.0)	1990 (31.3)
45–54	1089 (29.5)	705 (26.5)	1794 (28.2)
>55	367 (9.9)	155 (5.8)	522 (8.2)
*Mean age (±SD)*	40.6 (±10.6)	39.1 (±9.9)	40.1 (±10.3)
**Education**			
Illiterate	169 (4.6)	394 (14.8)	563 (8.9)
Literate, but no formal education	57 (1.5)	80 (3.0)	137 (2.2)
Primary school complete	352 (9.5)	477 (17.9)	829 (13.0)
Secondary and higher secondary complete	2017(54.5)	1074 (40.4)	3091 (48.6)
Graduate	788 (21.3)	420 (15.8)	1208 (19.0)
Professional degrees	315 (8.5)	214 (8.1)	529(8.3)
**Standard of living index** b			
Low	263 (7.1)	162 (6.1)	425 (6.7)
Medium	529 (14.3)	273 (10.3)	802 (12.6)
High	2906 (78.6)	2224 (83.6)	5130 (80.7)
**Migration status**			
Rural	1374 (40.5)	596 (24.3)	1970 (33.7)
Migrant	962 (28.3)	867 (35.3)	1829 (31.3)
Urban	1059 (31.2)	991 (40.4)	2050 (35.1)
**Tobacco consumption**			
Never	2177 (58.9)	2563 (96.4)	4740 (74.6)
Past	156 (4.2)	30 (1.1)	186 (2.9)
Current	1365 (36.9)	66 (2.5)	1431 22.5)
**Alcohol consumption**			
Never	2647 (71.6)	2528 (95.1)	5175(81.4)
Past	149 (4.0)	41 (1.5)	190 (3.0)
Current	902 (24.4)	90 (3.4)	992 (15.6)
**Energy expenditure (MET hours/day)** c			
Quartile I	760 (20.6)	816 (30.7)	1576 (24.8)
Quartile II	843 (22.8)	730 (27.5)	1573 (24.7)
Quartile III	931 (25.2)	644 (24.2)	1575 (24.8)
Quartile IV	1164 (31.5)	469 (17.6)	1633 (25.7)
**Energy intake (kcal)^d^**			
Quartile I	670 (18.1)	920 (34.6)	1590 (25.0)
Quartile II	795 (22.5)	794 (29.9)	1589 (25.0)
Quartile III	990 (26.8)	599 (22.5)	1589 (25.0)
Quartile IV	1243 (33.6)	346 (13.0)	1589 (25.0)

a Amount in one portion: 120 g for plain milk; 124 g for tea; 195 g for curd; 143 g for buttermilk/lassi.

b The standard of living index (SLI) is based on following assets in the household: quality of house; toilet facilities; source of lighting and drinking water; possession of clock, radio, television, bicycle, motorcycle, car, tractor, refrigerator, and telephone. These items were scored with a maximum score of 38. Scores were given according to the scores developed by the International Institute of Population Sciences in India (IIPS and ORC Macro 2000), and were based on a priori knowledge about the relative significance of the items. A score of less than 20 indicates low SLI, 21–26 indicates medium SLI, and 27 and above indicates high SLI.

c MET: Metabolic Equivalent of Task: Quartile I: <35.7084; Quartile II: 35.7084-38.2888; Quartile III: 38.2889-41.5417; Quartile IV : > 41.5418; ^d^ Quartile I: <2220.45; Quartile II: 2220.45-2781.45; Quartile III: 2781.46-3514.16; Quartile IV: > 3514.17.

### Milk/milk Product Consumption in the Population according to Selected Characteristics

Table 2 shows the daily plain milk, tea, curd and buttermilk/*lassi* consumption pattern in the sample population according to selected characteristics. More than half the population with age >55 years, 54% of the women population, more than two third of the illiterate population, population belonging to low standard of living households never drink milk. Drinking ≥1 portion of milk daily was more common in the age group below 25 years, among men, among those with graduate and professional degrees and in the high standard of living households, among those who were more physically active and among those whose energy intake was high. One in eleven participants never drank tea while four out of five drink ≥1 portion tea daily. Drinking ≥1 portion of tea daily was more common in age 35–44 years, among men, among those with a secondary and higher secondary complete education, who belonged to high standard of living households, and those who never consumed tobacco or alcohol. More than one out of five sample population never consumed curd, while 16% consumed ≥ 1 portion daily. Drinking ≥1 portion of curd daily was more common in age 45–54 years, in men, among those with a secondary and higher secondary complete education, who belonged to high standard of living households, and among those who never consumed tobacco or alcohol. One out of four participants never drink buttermilk/*lassi* while only 14% drink ≥ 1 portion daily. Other characteristics of the daily buttermilk/*lassi* drinkers were almost similar to the daily curd consumers.

**Table 2:** Daily plain milk, tea, curd, and buttermilk/*lassi* consumption pattern in the sample population according to selected background characteristics, lifestyle indicators, energy intake and physical activity, Indian Migration Study, 2006.

Characteristics	Daily plain milk consumption	Daily tea consumption	Daily curd consumption	Daily buttermilk/*lassi* consumption								
	Never	<1 portion	≥ 1 portion	Never	<1 portion	≥ 1 portion	Never	<1 portion	≥ 1 portion	Never	<1 portion	≥ 1 portion
	N[%]	N[%]	N[%]	N[%]	N[%]	N[%]	N[%]	N[%]	N[%]	N[%]	N[%]	N[%]
Total	3084[48.5]	1783[28.1]	1490[23.4]	567[8.9]	572[9.0]	5218[82.1]	1419[22.3]	3901[61.4]	1037[16.3]	1582[24.9]	3857[60.7]	918[14.4]
**Age (years)**		p<0.0001			p<0.0001			p<0.0001			p<0.0001	
18–25	137[30.6]	181[40.4]	130[29.0]	30[5.3]	89[15.6]	329[6.3]	67[4.7]	350[9.0]	31[3.0]	83[5.3]	350[9.1]	15[1.6]
25–34	689[43.0]	565[35.3]	349[21.8]	113[19.9]	155[27.1]	1335[25.6]	260[18.3]	1124[28.8]	219[21.1]	333[21.1]	1167[30.3]	103[11.2]
35–44	1026[51.6]	504[25.3]	460[23.1]	188[33.2]	139[24.3]	1663[31.9]	479[33.8]	1172[30.0]	339[32.7]	526[33.3]	1136[29.5]	328[35.7]
45–54	944[52.6]	411[22.9]	439[24.5]	177[31.2]	145[25.4]	1472[28.2]	466[32.8]	975[25.0]	353[34.0]	469[29.7]	956[24.8]	369[40.2]
>55	288[55.2]	122[23.4]	112[21.5]	59[10.4]	44[7.7]	419[8.0]	147[10.4]	280[7.2]	95[9.2]	171[10.8]	248[6.4]	103[11.2]
**Gender**		p<0.0001			p<0.0001			p<0.0001			p<0.0001	
Men	1645[44.5]	1095[29.6]	958[25.9]	254[44.8]	310[54.2]	3134[60.1]	778[54.8]	2259[57.9]	661[63.7]	835[52.8]	2356[61.1]	507[55.2]
Women	1439[54.1]	688[25.9]	532[20.0]	313[55.2]	262[45.8]	2084[39.9]	641[45.2]	1642[42.1]	376[36.3]	747[47.2]	1501[38.9]	411[44.8]
**Education**		p<0.0001			p = 0.009			p<0.0001			p<0.0001	
Illiterate	380[67.5]	110[19.5]	73[13.0]	59[10.4]	54[9.4]	450[8.6]	186[13.1]	300[7.7]	77[7.4]	293[18.5]	219[5.7]	51[5.6]
Literate, but no formal education	84[61.3]	31[22.6]	22[16.0]	13[2.3]	8[1.4]	116[2.2]	43[3.0]	80[2.1]	14[1.4]	52[3.3]	70[1.8]	15[1.6]
Primary school complete	471[56.8]	211[25.5]	147[17.7]	87[15.3]	69[12.1]	673[12.9]	245[17.3]	446[11.4]	138[13.3]	260[16.4]	399[10.3]	170[18.5]
Secondary and higher secondary complete	1447[46.8]	935[30.3]	709[22.9]	246[43.4]	306[53.5]	2539[48.7]	652[46.0]	1939[49.7]	500[48.2]	650[41.1]	2007[52.0]	434[47.3]
Graduate	485[40.2]	366[30.3]	357[30.0]	123[21.7]	104[18.2]	981[18.8]	211[14.9]	790[20.3]	207[20.0]	231[14.6]	794[20.6]	183[19.9]
Professional degrees	217[41.0]	130[24.6]	182[34.4]	39[6.9]	31[5.4]	459[8.8]	82[5.8]	346[8.9]	101[9.7]	96[6.1]	368[9.5]	65[7.1]
**Standard of living index**		p<0.0001			p<0.0001			p<0.0001			p<0.0001	
Low	282[66.4]	89[20.9]	54[12.7]	41[7.2]	60[10.5]	324[6.2]	167[11.8]	226[5.8]	32[3.1]	207[13.1]	175[4.5]	43[4.7]
Medium	381[47.5]	235[29.3]	186[23.2]	73[12.9]	93[16.3]	636[12.2]	229[16.1]	480[12.3]	93[9.0]	241[15.2]	468[12.1]	93[10.1]
High	2421[47.2]	1459[28.4]	1250[24.4]	453[79.9]	419[73.3]	4258[81.6]	1023[72.1]	3195[81.9]	912[88.0]	1134[71.7]	3214[83.3]	782[85.2]
**Migration status**		p = 0.017			p = 0.001			p<0.0001			p<0.0001	
Rural	892[45.3]	566[28.7]	512[26.0]	190[36.0]	202[38.9]	1578[32.9]	511[39.2]	1175[32.8]	284[29.6]	620[43.7]	1044[29.5]	306[34.5]
Migrant	915[50.0]	516[28.2]	398[21.8]	148[28.0]	175[33.7]	1506[31.4]	387[29.7]	1072[29.9]	370[38.5]	412[29.0]	1145[32.3]	272[30.7]
Urban	993[48.4]	572[27.9]	485[23.7]	190[36.0]	142[27.4]	1718[35.8]	406[31.1]	1338[37.3]	306[31.9]	387[27.3]	1355[38.2]	308[34.8]
**Tobacco consumption**		p = 0.014			p<0.0001			p = 0.057			p<0.0001	
Never	2256[47.6]	1348[28.4]	1136[24.0]	504[88.9]	457[79.9]	3779[72.4]	1095[77.2]	2874[73.7]	771[74.4]	1172[74.1]	2805[72.7]	763[83.1]
Past	111[59.7]	41[22.0]	34[18.3]	15[2.7]	20[3.5]	151[2.9]	35[2.5]	113[2.9]	38[3.7]	48[3.0]	105[2.7]	33[3.6]
Current	717[50.1]	394[27.5]	320[22.4]	48[8.5]	95[16.6]	1288[24.7]	289[30.4]	914[23.4]	228[22.0]	362[22.9]	947[24.6]	122[13.3]
**Alcohol consumption**		p<0.0001			p<0.0001			p<0.0001			p<0.0001	
Never	2368[45.8]	1508[29.1]	1299[25.1]	505[89.1]	477[83.4]	4193[80.4]	1129[79.6]	3254[83.4]	792[76.4]	1207[76.3]	3197[82.9]	771[84.0]
Past	103[54.2]	48[25.3]	39[20.5]	15[2.7]	15[2.6]	160[3.1]	48[3.4]	102[2.6]	40[3.9]	54[3.4]	106[2.8]	30[3.3]
Current	613[61.8]	227[22.9]	152[15.3]	47[8.3]	80[14.0]	865[16.6]	242[17.1]	545[14.0]	205[19.8]	321[20.3]	554[14.4]	117[12.8]
**Energy expenditure** **(MET hours/day)**		p<0.0001			p = 0.010			p<0.0001			p<0.0001	
Quartile I	845[53.6]	408[25.9]	323[20.5]	146[25.8]	123[21.5]	1307[25.1]	456[32.1]	859[22.0]	261[25.2]	444[28.1]	891[23.1]	241[26.3]
Quartile II	747[47.5]	446[28.4]	380[24.2]	133[23.5]	130[22.7]	1310[25.1]	312[22.0]	1010[25.9]	251[24.2]	361[22.8]	997[25.9]	215[23.4]
Quartile III	754[47.9]	434[27.6]	387[24.6]	147[25.9]	133[23.3]	1295[24.8]	306[21.6]	986[25.3]	283[27.3]	352[22.3]	956[25.0]	258[28.1]
Quartile IV	738[45.2]	495[30.3]	400[24.5]	141[24.9]	186[32.5]	1306[25.0]	345[34.3]	1046[26.8]	242[23.3]	425[26.9]	1004[26.0]	204[22.2]
**Energy intake (kcal)**		p<0.0001			p<0.0001			p<0.0001			p<0.0001	
Quartile I	976[61.4]	360[22.6]	254[16.0]	205[36.2]	197[34.4]	1188[22.8]	596[42.0]	854[21.9]	140[13.5]	681[43.1]	730[18.9]	179[19.5]
Quartile II	812[51.1]	409[25.7]	368[23.2]	156[27.5]	161[28.2]	1272[24.4]	404[28.5]	952[24.4]	233[22.5]	372[23.5]	963[25.0]	254[27.7]
Quartile III	719[45.3]	464[29.2]	406[25.6]	115[20.3]	115[20.1]	1359[26.0]	251[17.7]	1072[27.5]	266[25.7]	278[17.6]	1095[28.4]	216[23.5]
Quartile IV	577[36.3]	550[34.6]	462[29.1]	91[16.1]	99[17.3]	1399[26.8]	168[11.8]	1023[26.2]	398[38.4]	251[15.9]	1069[27.7]	269[29.3]

Note: p values are from chi square test of significance.

### Association of milk/milk Product Consumption and Socio-demographic and Lifestyle Variables with Anthropometric Indicators

Table 3 shows percentage prevalence of anthropometric indicators in the categories of milk and milk product consumption and as well as in the other socio-demographic and lifestyle variable categories. Daily consumption of plain milk was associated with lower prevalence of obesity among men (p<0.0001), as well as with lower WC among women (p = 0.005). The opposite trend was observed for tea, curd and buttermilk/*lassi* consumption, with increased consumption being associated with a higher prevalence of obesity and WC (p<0.0001) in both the sexes. Anthropometric measures also tend to increase with age, education and standard of living index (p<0.0001). Substantial differences across place of residence as well as between the rural, migrant and urban groups were also observed for both men and women (p<0.0001). Anthropometric indicators also tended to decrease with tobacco consumption, increase with alcohol consumption and was highest in the middle two quartiles of energy intake among men (p<0.0001), and decrease with increasing quartiles of physical activity among both men and women (p = <0.0001).

**Table 3:** Percentage distribution of anthropometric indicators among Men (n = 3,698) and Women (n = 2,659) according to milk/milk product consumption and other selected characteristics, Indian Migration Study, 2006.

Characteristics	Body Mass Index ≥25 kg/m^2^	Waist Circumference						
	Men	^2^p value	Women	^2^p value	Men (>90 cm)	^2^p value	Women (>80 cm)	^2^p value
	N (%)		N (%)		N (%)		N (%)	
**Daily plain milk consumption**		<0.0001		<0.0001		<0.0001		0.005
Never	572 (34.8)		678 (47.1)		612 (37.2)		652 (45.3)	
< 1 portion	287 (6.2)		258 (37.5)		281 (25.7)		266 (38.7)	
≥ 1 portion	265 (27.7)		205 (38.5)		318 (33.2)		211 (40.0)	
**Daily tea consumption**		<0.0001		0.005		0.001		0.014
Never	70 (27.6)		146 (46.7)		74 (29.1)		137 (43.8)	
< 1 portion	61 (19.7)		89 (34.0)		75 (24.2)		89 (34.0)	
≥ 1 portion	992 (31.7)		903 (43.4)		1062 (33.9)		903 (43.3)	
**Daily curd consumption**		<0.0001		<0.0001		<0.0001		<0.0001
Never	263 (33.8)		307 (47.9)		284 (36.5)		303 (47.3)	
< 1 portion	616 (27.3)		631 (38.4)		679 (30.1)		631 (38.4)	
≥ 1 portion	245 (37.1)		203 (54.0)		248 (37.5)		195 (51.9)	
**Daily buttermilk/** ***lassi*** ** consumption**		<0.0001		<0.0001		<0.0001		<0.0001
Never	226 (27.1)		324 (43.4)		255 (30.5)		317 (42.4)	
< 1 portion	705 (29.9)		586 (39.0)		743 (31.5)		592 (39.4)	
≥ 1 portion	193 (38.1)		231 (56.2)		213 (42.0)		220 (53.5)	
**Age (years)**		<0.0001		<0.0001		<0.0001		<0.0001
18–25	12 (5.3)		8 (3.6)		8 (3.5)		21 (9.6)	
25–34	167 (17.0)		149 (24.0)		117 (11.9)		155 (24.9)	
35–44	348 (33.7)		497 (51.9)		350 (33.9)		456 (47.7)	
45–54	479 (44.0)		416 (59.0)		566 (52.0)		416 (59.0)	
>55	118 (32.2)		71 (45.8)		170 (46.3)		81 (52.3)	
**Education**		<0.0001		<0.0001		<0.0001		0.002
Illiterate	27 (16.0)		132 (33.5)		28 (16.6)		147 (37.3)	
Literate, but no formaleducation	13 (22.8)		32 (40.0)		16 (28.1)		38 (47.5)	
Primary schoolcomplete	70 (19.9)		238 (49.9)		83 (23.6)		234 (49.1)	
Secondary and highersecondary complete	600 (29.8)		436 (40.6)		618 (30.6)		435 (40.5)	
Graduate	284 (36.0)		189 (45.0)		318 (40.4)		172 (41.0)	
Professional degrees	130 (41.3)		114 (53.3)		148 (47.0)		103 (48.1)	
**Standard of living index**		<0.0001		<0.0001		<0.0001		<0.0001
Low	17 (6.5)		26 (16.1)		24 (9.1)		35 (21.6)	
Medium	64 (12.1)		55 (20.2)		62 (11.7)		64 (23.4)	
High	1043 (35.9)		1060 (47.7)		1125 (38.7)		1030 (46.3)	
**Migration status**		<0.0001		<0.0001		<0.0001		<0.0001
Rural	236 (17.2)		158 (26.5)		270 (19.7)		181 (30.4)	
Migrant	389 (40.4)		417 (48.1)		429 (44.6)		435 (50.2)	
Urban	429 (40.5)		509 (51.4)		437 (41.3)		455 (45.9)	
**Tobacco consumption**		<0.0001		0.155		<0.0001		0.581
Never	718 (33.0)		1109 (43.3)		781 (36.9)		1090 (42.5)	
Past	57 (36.5)		10 (33.3)		60 (38.5)		10 (33.3)	
Current	349 (25.6)		22 (33.3)		370 (27.1)		29 (43.9)	
**Alcohol consumption**		0.015		0.086		0.001		0.788
Never	769 (29.1)		1086 (43.0)		818 (30.9)		1074 (42.5)	
Past	54 (36.2)		23 (56.1)		52 (34.9)		19 (46.3)	
Current	301 (33.4)		32 (35.6)		341 (37.8)		36 (40.0)	
**Energy expenditure** **(MET hours/day)**		<0.0001		<0.0001		<0.0001		<0.0001
Quartile I	163 (24.3)		370 (40.2)		189 (28.2)		377 (41.0)	
Quartile II	246 (30.9)		377 (47.5)		279 (35.1)		371 (46.7)	
Quartile III	332 (33.5)		248 (41.4)		378 (38.2)		243 (40.6)	
Quartile IV	383 (30.8)		146 (42.2)		365 (29.4)		138 (40.0)	
**Energy intake (kcal)**		0.001		0.018		<0.0001		0.036
Quartile I	296 (39.0)		381 (46.7)		314 (41.3)		390 (47.8)	
Quartile II	294 (31.6)		321 (44.0)		334 (39.6)		313 (42.9)	
Quartile III	230 (19.8)		285 (44.3)		325 (34.9)		273 (42.4)	
Quartile IV	245 (23.0)		154 (32.8)		238 (20.5)		153 (32.6)	

### Association of Milk/milk Product Consumption and Anthropometric Indicators

Tables 4 show the effects of daily plain milk, tea, curd, and buttermilk/*lassi* consumption on BMI, and WC among men and women in four different models. The unadjusted odds (Model 1) of having a high BMI (≥25 kg/m^2^) were lower among men and women who consumed ≥1 portion of plain milk daily (men: OR = 0.66;95%CI,0.53−0.82;p<0.0001; women: OR = 0.63; 95%CI, 0.48−0.83; p = 0.001) than those who never consumed plain milk. Controlling for socioeconomic and demographic factors in Model 2, behavioural factors such as tobacco consumption, alcohol consumption, physical activity (MET hours/day) and energy intake (kcal) in Model 3 and all potential confounders together in Model 4 did not attenuate the strong inverse association between daily consumption of ≥1 portion of plain milk and the risk of having a high BMI.

**Table 4:** Unadjusted and adjusted association (ORs with 95%CI) of milk/milk product consumption on anthropometric indicators for men (n = 3,698) and women (n = 2,659), Indian Migration Study, 2006.

Characteristics	Model 1 Unadjusted^a^	Model 2 Adjusted^b^	Model 3 Adjusted^c^	Model 4 Adjusted^d^				
	BMI^e^	WC^f^	BMI	WC	BMI	WC	BMI	WC
	OR[95%CI]	OR[95%CI]	OR[95%CI]	OR[95%CI]	OR[95%CI]	OR[95%CI]	OR[95%CI]	OR[95%CI]
**MEN**								
Daily plain milk consumption								
Never	1.00[ref]	1.00[ref]	1.00[ref]	1.00[ref]	1.00[ref]	1.00[ref]	1.00[ref]	1.00[ref]
< 1 portion	0.61[0.50–0.76]	0.53[0.41–0.68]	0.72[0.56–0.92]	0.69[0.53–0.89]	0.61[0.47–0.76]	0.56[0.41–0.72]	0.71[0.55–0.91]	0.68[0.52–0.88]
≥ 1 portion	0.66[0.53–0.82]	0.77[0.59–0.99]	0.69[0.53–0.90]	0.72[0.55–0.93]	0.65[0.50–0.83]	0.82[0.63–1.09]	0.67[0.51–0.87]	0.71[0.54–0.93]
Daily tea consumption								
Never	1.00[ref]	1.00[ref]	1.00[ref]	1.00[ref]	1.00[ref]	1.00[ref]	1.00[ref]	1.00[ref]
< 1 portion	0.63[0.39–1.00]	0.84[0.52–1.34]	0.78[0.45–1.34]	1.19[0.70–2.05]	0.70[0.43–1.15]	0.97[0.59–1.60]	0.81[0.46–1.41]	1.22[0.71–2.12]
≥ 1 portion	1.39[0.97–1.97]	1.53[1.06–2.20]	1.49[0.99–2.22]	1.66[1.10–2.50]	1.46[1.00–2.12]	1.66[1.13–2.44]	1.51[1.00–2.28]	1.65[1.08–2.51]
Daily curd consumption								
Never	1.00[ref]	1.00[ref]	1.00[ref]	1.00[ref]	1.00[ref]	1.00[ref]	1.00[ref]	1.00[ref]
< 1 portion	0.72[0.58–0.90]	0.80[0.62–1.04]	0.78[0.60–1.01]	0.77[0.59–1.00]	0.71[0.54–0.89]	0.72[0.57–0.92]	0.73[0.55–0.96]	0.71[0.51–0.94]
≥ 1 portion	1.25[0.95–1.63]	1.01[0.73–1.41]	1.03[0.74–1.42]	0.99[0.72–1.36]	1.09[0.82–1.47]	1.06[0.78–1.44]	0.93[0.66–1.30]	0.91[0.65–1.27]
Daily buttermilk/*lassi* consumption								
Never	1.00[ref]	1.00[ref]	1.00[ref]	1.00[ref]	1.00[ref]	1.00[ref]	1.00[ref]	1.00[ref]
< 1 portion	1.23[0.99–1.54]	1.32[1.01–1.73]	1.15[0.88–1.49]	0.99[0.76–1.82]	1.15[0.91–1.46]	1.06[0.84–1.35]	1.11[0.85–1.46]	0.95[0.73–1.25]
≥ 1 portion	1.89[1.40–2.55]	1.90[1.32–2.72]	1.01[0.70–1.45]	1.03[0.72–1.47]	1.59[1.15–2.19]	1.68[1.21–2.33]	0.95[0.65–1.38]	0.98[0.68–1.42]
**WOMEN**								
Daily plain milk consumption								
Never	1.00[ref]	1.00[ref]	1.00[ref]	1.00[ref]	1.00[ref]	1.00[ref]	1.00[ref]	1.00[ref]
<1 portion	0.61[0.47–0.78]	0.80[0.59–1.07]	0.81[0.62–1.05]	0.93[0.72–1.20]	0.66[0.50–0.83]	0.71[0.56–0.90]	0.81[0.62–1.06]	0.94[0.72–1.21]
≥1 portion	0.63[0.48–0.83]	0.70[0.50–0.98]	0.58[0.43–0.77]	0.78[0.59–1.04]	0.65[0.48–0.83]	0.75[0.58–0.97]	0.57[0.43–0.76]	0.79[0.59–1.05]
Daily tea consumption								
Never	1.00[ref]	1.00[ref]	1.00[ref]	1.00[ref]	1.00[ref]	1.00[ref]	1.00[ref]	1.00[ref]
< 1 portion	0.54[0.35–0.84]	0.64[0.42–0.97]	1.15[0.72–1.85]	1.17[0.73–1.86]	0.54[0.35–0.85]	0.64[0.42–0.97]	1.13[0.71–1.80]	1.15[0.73–1.84]
≥ 1 portion	0.89[0.65–1.22]	1.02[0.75–1.38]	1.26[0.91–1.74]	1.23[0.88–1.72]	0.86[0.62–1.18]	0.99[0.73–1.34]	1.25[0.90–1.74]	1.22[0.87–1.70]
Daily curd consumption								
Never	1.00[ref]	1.00[ref]	1.00[ref]	1.00[ref]	1.00[ref]	1.00[ref]	1.00[ref]	1.00[ref]
<1 portion	0.64[0.50–0.81]	0.64[0.48–0.85]	0.83[0.64–1.09]	0.78[0.60–1.02]	0.61[0.48–0.79]	0.68[0.54–0.86]	0.83[0.63–1.09]	0.79[0.60–1.02]
≥1 portion	1.34[0.96–1.88]	1.03[0.70–1.51]	0.82[0.59–1.19]	0.73[0.51–1.04]	1.25[0.89–1.77]	1.21[0.88–1.69]	0.81[0.56–1.17]	0.72[0.43–1.05]
Daily buttermilk/*lassi* consumption								
Never	1.00[ref]	1.00[ref]	1.00[ref]	1.00[ref]	1.00[ref]	1.00[ref]	1.00[ref]	1.00[ref]
<1 portion	0.83[0.66–1.05]	0.79[0.60–1.05]	1.02[0.79–1.32]	1.03[0.75–1.43]	0.78[0.61–0.99]	0.87[0.69–1.10]	1.08[0.83–1.41]	1.04[0.75–1.44]
≥1 portion	1.84[1.34–2.54]	1.13[0.78–1.66]	1.03[0.73–1.45]	1.23[0.80–1.90]	1.76[1.27–2.44]	1.69[1.24–2.31]	1.10[0.78–1.57]	1.25[0.82–1.73]

a Model 1 is unadjusted using mixed-effect logistic regression analysis.

b Model 2 is adjusted for socioeconomic and demographic characteristics: age, education, standard of living, migration status with an individual-specific random effect of sib-pair.

c Model 3 is adjusted for behavioral factors: tobacco consumption, alcohol consumption, energy expenditure and energy intake with an individual-specific random effect of sib-pair.

d Model 4 is adjusted for all the above factors.

e Dependent variable BMI: 0 ≤ 25 kg/m^2^ and 1 > 25 kg/m^2^.

f Dependent variable Men WC: 0 ≤ 90 cm & 1 >90 cm; Women WC: 0 ≤ 80 cm & 1 > 80 cm.

A similar pattern of associations between WC and plain milk consumption was found for men and women although the relationship was less strong and the evidence of association was weak after adjustment for confounding factors, particularly among women.

Tea consumption of ≥1 portion/ day was associated with obesity (OR = 1.51;95%CI:1.00−2.25;p>0.050) and high WC (OR = 1.65;95%CI:1.08−2.51;p>0.019) among men but non-significant positive association (obesity OR = 1.25;95%CI:0.90−1.74; WC (OR = 1.22;95%CI:0.87−1.70) was observed in case of women in the fully adjusted model. A strong evidence of association was constantly observed between ≥1 portion/ daily tea consumption and high WC all through the unadjusted and the adjusted models in men (Table 4).

Daily curd consumption did not have an effect on the risk for an adverse anthropometric profile among men or women in either unadjusted or adjusted models. Buttermilk/*lassi* consumption did seem to have an effect in men though statistically not significant, but the association is in the opposite direction to that of plain milk consumption in case of women. Unadjusted analysis in Model 1 shows daily consumption of ≥1 portion of buttermilk/*lassi* to increase the risk for an adverse anthropometric profile by almost two times among men (BMI [OR = 1.89; 95%CI, 1.40−2.55; p<0.0001]; WC [OR = 1.90; 95%CI, 1.32−2.72; p = 0.001]) and these effects remain almost unchanged when behavioural factors are additionally controlled for in Model 3 (BMI [OR = 1.59; 95%CI, 1.15−2.19; p = 0.003]; WC [OR = 1.68; 95%CI, 1.21−2.33; p = 0.002]). However, these effects are markedly attenuated when socioeconomic and demographic factors are adjusted in Model 2 and when all the potential confounders were collectively controlled for in Model 4. In women an opposite trend to that of plain milk consumption in seen between buttermilk/*lassi*, BMI as well as WC.

In analyses taking account of the sibling-pair data structure (Table 5), the inverse association between milk consumption and BMI for men and women were similar in between-sibling-pairs and within-sibling-pairs, with no statistical evidence of differences between the effects (Table 5). The findings were also similar for WC.

**Table 5:** Within and between sib-pair effects of milk/milk product consumption on obesity and waist circumference among men (n = 3698) and women (n = 2659) (Estimates from a population-average approach).

Outcome measures	Model 1 Unadjusted^a^	Model 2 Adjusted^b^	Model 3 Adjusted^c^	Model 4 Adjusted^d^				
	OR	[95%CI]	OR	[95%CI]	OR	[95%CI]	OR	[95%CI]
**MEN**								
**Obesity (BMI>25 kg/m^2^)**								
Plain milk within	0.84	[0.71–1.00]	0.90	[0.74–1.09]	0.82	[0.70–0.99]	0.89	[0.73–1.08]
Plain milk between	0.82	[0.74–0.91]	0.85	[0.75–0.95]	0.81	[0.73–0.90]	0.83	[0.73–0.93]
Test within = between	0.7864		0.6017		0.7970		0.5498	
Tea within	1.53	[1.21–1.93]	1.43	[1.10–1.87]	1.51	[1.19–1.90]	1.40	[1.08–1.83]
Tea between	1.17	[1.00–1.37]	1.18	[0.99–1.41]	1.16	[0.99–1.36]	1.18	[0.98–1.41]
Test within = between	0.0530		0.2188		0.0619		0.2690	
Curd within	1.29	[1.05–1.59]	1.14	[0.90–1.44]	1.11	[0.97–1.47]	1.11	[0.88–1.41]
Curd between	1.00	[0.88–1.15]	0.94	[0.81–1.09]	0.95	[0.84–1.11]	0.91	[0.78–1.06]
Test within = between	0.0441		0.1776		0.0823		0.1606	
Buttermilk/*lassi* within	1.32	[1.03–1.68]	1.04	[0.79–1.36]	1.19	[0.92–1.51]	1.02	[0.78–1.34]
Buttermilk/*lassi* between	1.27	[1.11–1.45]	1.01	[0.86–1.19]	1.17	[1.03–1.37]	1.02	[0.86–1.21]
Test within = between	0.7925		0.8438		0.9516		0.9986	
**Waist circumference (>90cm)**								
Plain milk within	0.87	[0.75–1.02]	0.90	[0.73–1.09]	0.87	[0.74–1.02]	0.89	[0.73–1.09]
Plain milk between	0.91	[0.83–1.00]	0.90	[0.80–1.02]	0.94	[0.85–1.04]	0.89	[0.78–1.01]
Test within = between	0.6281		0.9587		0.9260		0.9820	
Tea within	1.63	[1.31–2.04]	1.58	[1.20–2.07]	1.63	[1.30–2.04]	1.54	[1.18–2.02]
Tea between	1.11	[0.95–1.30]	1.12	[0.93–1.33]	1.11	[0.95–1.30]	1.11	[0.93–1.33]
Test within = between	0.0039		0.0324		0.0049		0.0447	
Curd within	1.32	[1.09–1.60]	1.19	[0.93–1.51]	1.27	[1.04–1.54]	1.16	[0.90–1.48]
Curd between	0.98	[0.86–1.12]	0.95	[0.81–1.11]	0.97	[0.85–1.11]	0.92	[0.78–1.08]
Test within = between	0.0110		0.1304		0.0264		0.1263	
Buttermilk/*lassi* within	1.50	[1.20–1.88]	1.14	[0.86–1.50]	1.35	[1.07–1.70]	1.10	[0.83–1.46]
Buttermilk/*lassi* between	1.26	[1.11–1.44]	1.05	[0.89–1.24]	1.21	[1.06–1.39]	1.06	[0.90–1.26]
Test within = between	0.1950		0.6362		0.4247		0.8248	
**WOMEN**								
**Obesity (BMI>25 kg/m^2^)**								
Daily plain milk within	0.78	[0.64–0.96]	0.74	[0.57–0.96]	0.76	[0.62–0.94]	0.73	[0.56–0.95]
Daily plain milk between	0.83	[0.74–0.92]	0.81	[0.70–0.92]	0.82	[0.72–0.90]	0.81	[0.70–0.92]
Test within = between	0.6443		0.5412		0.6856		0.5295	
Tea within	1.16	[0.93–1.46]	1.15	[0.86–1.55]	1.15	[0.91–1.44]	1.14	[0.85–1.54]
Tea between	0.97	[0.85–1.10]	1.09	[0.94–1.27]	0.95	[0.83–1.09]	1.08	[0.92–1.27]
Test within = between	0.1665		0.7495		0.1639		0.7621	
Curd within	1.02	[0.79–1.33]	0.84	[0.60–1.16]	1.02	[0.78–1.33]	0.83	[0.60–1.17]
Curd between	1.04	[0.90–1.20]	0.93	[0.79–1.10]	1.01	[0.87–1.17]	0.93	[0.78–1.11]
Test within = between	0.9027		0.5666		0.9698		0.5792	
Buttermilk/*lassi* within	1.07	[0.81–1.40]	0.96	[0.69–1.33]	1.04	[0.79–1.37]	0.96	[0.69–1.34]
Buttermilk/*lassi* between	1.22	[0.07–1.40]	1.03	[0.88–1.21]	1.21	[1.05–1.39]	1.07	[0.91–1.26]
Test within = between	0.3778		0.7027		0.3299		0.5758	
**Waist circumference (>80cm)**								
Plain milk within	1.21	[0.92–1.59]	1.25	[0.92–1.69]	1.18	[0.89–1.56]	1.24	[0.91–1.69]
Plain milk between	0.78	[0.68–0.91]	0.85	[0.72–1.00]	0.80	[0.68–0.93]	0.85	[0.72–1.00]
Test within = between	0.0075		0.0281		0.0162		0.0365	
Tea within	1.21	[0.96–1.53]	1.20	[0.90–1.59]	1.19	[0.94–1.51]	1.19	[0.89–1.58]
Tea between	1.02	[0.89–1.16]	1.06	[0.91–1.23]	1.01	[0.88–1.15]	1.05	[0.90–1.23]
Test within = between	0.2047		0.4461		0.2230		0.4712	
Curd within	1.10	[0.78–1.57]	0.95	[0.66–1.37]	1.16	[0.81–1.66]	0.95	[0.65–1.39]
Curd between	0.91	[0.76–1.10]	0.82	[0.68–0.99]	0.93	[0.77–1.12]	0.81	[0.66–0.99]
Test within = between	0.3520		0.4952		0.2804		0.4377	
Buttermilk/*lassi* within	1.13	[0.79–1.62]	1.04	[0.72–1.52]	1.12	[0.78–1.62]	1.08	[0.74–1.58]
Buttermilk/*lassi* between	0.99	[0.84–1.17]	1.04	[0.82–1.26]	1.03	[0.87–1.23]	1.09	[0.90–1.33]
Test within = between	0.5065		0.9928		0.6797		0.9524	

a Model 1 is unadjusted.

b Model 2 is adjusted for socioeconomic and demographic characteristics: age, education, standard of living, and migration status.

c Model 3 is adjusted for behavioral factors: tobacco consumption, alcohol consumption, MET hours/day and energy intake.

d Model 4 is adjusted for all the above factors.

## Discussion

The present study demonstrates evidence of a substantial inverse association of daily plain milk consumption with the risk of being obese in a large sample of adult men and women in India. This association, found for BMI and WC in men and women, is independent of age, education, standard of living, migration status, alcohol intake, tobacco consumption, energy intake and physical activity levels in both sexes. High tea consumption daily was associated with increased risk of obesity and high WC in men but not among women. There was no strong evidence of associations between daily curd or buttermilk/*lassi* consumption with BMI or WC in either men or women.

### Mechanisms

It is possible that higher dairy product consumption is associated with an overall healthy diet and lifestyle, which may track through the life course and ultimately lead to lower weight gain in adults [51]. Our analyses, adjusting for physical activity and several life-style characteristics, provide limited evidence against this mechanism. Milk is a source of high-quality protein and other nutrients, including lactose, proteins, fats, calcium, biotin, iodine, magnesium, pantothenic acid, potassium, riboflavin, selenium, thiamine, vitamin A, vitamin B_12_ and vitamin D [52] which may be implicated in metabolic pathways relevant to obesity. Two major biological mechanisms – body weight regulation and appetite regulation - for explaining the effects of dairy products have been proposed with some evidence to support roles for calcium, linoleic acid, medium chain fatty acids, milk proteins, carbohydrates and fats [53]. The precise biological and molecular mechanisms of action of dairy products remain to be fully elucidated.

### Results in Context of Other Studies

The results of this study are in line with other observational epidemiologic studies where an inverse association has been found between dairy consumption and risk of being overweight or obese. A recent study in India [54] showed that consumption of plain milk was higher among normal-weight than among obese individuals (p<0.05). Normal-weight individuals consumed 1.7-times more whole milk than did obese individuals, while the latter consumed 1.5-times more skimmed milk and 2.6-times more toned milk [54]. An inverse association between milk drinking and body weight or BMI is shown in the cross-sectional baseline data of a number of large cohort studies, [55]–[58] all from developed countries. For example, an association between dairy food consumption and body fat was found in the NHANES III survey data, in which the odds ratio of a subject whose milk intake was in the highest quartile, compared to the lowest quartile, was 0.16 (0.03–0.88) [59]. In a limited overview of six observational studies and three controlled trials [60] a consistent negative effect of calcium intake on body fat and body weight was reported and the authors estimated that each 300 mg increment in habitual dietary calcium intake (equivalent to an average portion of dairy food) is associated with ∼1 kg less body fat in children and ∼2.5–3.0 lower body weight in adults [61]. Unsurprisingly, there have been few relevant randomized trials assessing this question owing to difficulties in conducting long-term dietary intervention trials. Those that have been conducted were typically of less than 1 year duration and found no effects on changes in weight [53]. In one trial weight loss was greater in participants randomized to dairy, compared with those receiving equivalent amounts of calcium as a supplement or those on a low calcium diet [62].

We found daily consumption of more than one portion of tea is associated with increased risk of obesity and high WC among men but not among women in our study. However, this association attenuates when we do a gender adjusted analysis. The tea – obesity association is almost certainly due to the sugar content of the tea and drinking tea with sugar and milk is almost universal practice among Indians. Our plain milk variable does not include milk taken as tea, but when we added this consumption to give a ‘total milk’ consumption variable (combining plain milk, curd, buttermilk and tea), we still got an inverse association with obesity in the total population but the association was not significant. However, studies on tea consumption and obesity risk mostly focused on green and other tea (black, herbal etc.) consumption generally without milk [63]–[65] which have been linked to low weight gain and thus studies to confirm our finding are lacking.

Contrary to popular belief, in this study we did not find low-fat dairy products such as curd and buttermilk/*lassi* to be more beneficial to weight status than regular-fat dairy products such as plain milk. Our study findings are in line similar to other studies which suggest the reverse may be true. The weight of evidence from a recent review [66] suggested that milk intake was more likely to be associated with beneficial weight outcomes in a number of studies [67]–[71], while a very small number of studies [72] showed beneficial effects of other dairy products such as yoghurt/cheese among subjects with specific baseline characteristics. In our study, milk and curd consumption were associated (chi2, 4 dof, 177.9, p<0005) but the pattern of association did not suggest that curd or buttermilk/*lassi* was substituted for milk in those taking no milk or vice versa. Though buttermilk and curds are similar in their origin, they do differ in composition and benefits. There is a regional variation in the consumption patterns of these two popular milk products in India. Buttermilk*/lassi* is mostly consumed in north India while curd in south India. Buttermilk is for drinking only and can be consumed as a whole, but curd is normally consumed additionally along with other food items in India, less frequently alone and thus required us to study these (plain milk, curd and buttermilk) separately because of their geographical variability in consumption and the method of preparation and consumption pattern in India.

### Strengths and Limitations

One of the strengths of the study is that analysis was confined to the healthy population of the sample, therefore minimizing errors associated with underlying disease conditions that may influence people to change their food habits. In addition, the study sample was large, representing populations from the northern, central and southern regions of India, although larger population wide studies will be needed before these results can be generalized to the whole country.

Unlike other observational studies we were able to make use of the sibling pair design of the Indian Migration Study to adjust for confounding in addition to the adjustments made in our conventional logistic regressions. The sibling pair design is beneficial because it provided a high level of control for potential confounding factors and for shared early life exposures as sib-pairs share many measured and unmeasured characteristics in common. Consistency of findings of analyses between groups and within sib-pairs makes it less likely that they are spurious and due to uncontrolled confounding. However, the requirement of siblings to travel to the factories to participate in the study created a high responder burden, and the response rate of 50% could have introduced selection bias, although previous analyses comparing those who took part with those who did not take part found no strong evidence of difference in health status [33]. Response bias would exaggerate differences seen in dietary intake if there was differential non-response whereby rural participants with the most different diets to their migrant siblings were more likely to participate [40].

Dietary information from a self-reported food frequency questionnaire has substantial error, including error in the estimation of portion sizes among individuals and the total number of foods actually consumed [73]. FFQs are useful for ascertaining the types of foods consumed, but are not as useful in assessing calorific intake [54]. But an FFQ was the only feasible method for measuring intake given that the study design required rural participants to travel on the days prior to assessment. The validity study found that the FFQ overestimated energy intake by 409 kcal compared to 24 hr recalls. However, overestimation of energy intake is a common finding in FFQs, and was also found in validation studies for region-specific FFQs conducted in India [74]–[76], [44].

Although data were collected from rural, urban as well as migrant groups, our analysis did not account for possible dietary differences between these groups. Moreover, data on family history would have informed us about the role of heritability together with food consumption habits in the manifestation of obesity. However, efforts in India to establish genetic associations with obesity to date have omitted the exploration of environmental factors [77] and evidence suggests that these novel genetic variants only modestly explain the variation in human BMI and body weight, which again points towards the important role of the environmental component [78]. Our sib-pair analysis – demonstrating similar findings to the main analysis – provides some control genetic variants associated with obesity and this was presented in a study by Taylor et al [79] with the IMS data set.

The participants in our study were sampled from factories and were therefore better off and more likely to be in stable employment than the Indian population in general. As well as being wealthier (around 80% have a high SLI score) than other population, the IMS migrant group worked in factories that provide housing and canteen facilities, and this may have accelerated the process of acculturation to urban lifestyle and diets, including levels of dairy consumption.

We found higher rates of obesity in our sample population which is in contrary to other Indian estimates of overweight and obesity (recent data [80] from India showed overweight/obesity is 12.6% in women and 9.7% in men). The possible explanation for this might be because more than 66% of our sample population were either rural-urban migrant or urban dwellers and studies showed that migration was associated with both an increased fat intake and reduced physical activity in both men and women, as compared with rural dwellers, and this likely contributes to the higher levels of obesity observed in migrants [33]. Also, in comparisons with the 3rd National Family Health Survey [80] and the 2001 Census of India [81], our study population had lower proportions of illiterate individuals and higher proportions of individuals with access to household facilities and assets, indicating a generally wealthier and more educated population than the national average in both rural and urban areas. It is a still paradox in India that overweight and obesity is positively associated with higher education (23.8% and 18.8% overweight/obesity in women and men respectively with 12 or more years of education in comparison to the illiterates-7.3% in women and 3.6% in men) and higher wealth status (30.5% and 23.6% overweight/obesity in women and men respectively in highest wealth category in comparison to lowest wealth category −1.8% in women and 1.4% in men) in the population [80].

Like all cross sectional studies, the data from this study was also prone to recall bias and despite use of a sib-pair design it is possible that residual confounding occurred and results should be interpreted with caution. Although physical activity and alcohol/tobacco consumption were controlled for, which further strengthens the negative association observed between milk intake and obesity, it does not exclude the possibility that milk intake may have been a proxy indicator of a generally healthier dietary pattern or lifestyle in this population.

The validity of the adjustment of association models for total energy intake needs to be carefully considered when interpreting study results and conducting cross-study comparisons. Given the high degree of correlation between total energy intake and adiposity, adjustment for total energy intake appears appropriate [82]. However, given that the beneficial impact of dairy constituents may be through an impact on appetite and food intake and therefore energy regulation, the adjustment for total energy intake may be misleading due to correction of the model for the mediator of the effect [53]. The associations we found with daily milk consumption were resistant to adjusted for energy intake, suggesting that this mechanism did not explain our findings.

### Implications of the Study Findings

Our finding in a large, sample of Indian adults from across the country, in which information on dietary patterns was captured using a valid and reliable FFQ developed for the Indian context does have implications for future research in India. Few studies have been conducted in developing countries where dairy consumption is increasing. Replication of our findings in other south Asian populations, particularly those living in high income settings would be useful because patterns of confounding will differ from those observed in this study of low to middle income Indians in India. Longitudinal changes in obesity-related indicators with intake of different kinds of milk, including low and high fat products, could be carried out, together with repeated and better assessments of all dairy components of the diet. Mendelian randomization studies using the lactase persistence gene might also be helpful in teasing out the unconfounded effect of milk consumption on obesity [83]. Attempts to conduct a Mendelian randomization study were thwarted as the lactase persistence gene was not associated with milk consumption in a large sample of British women [84]. In other populations, the lactase persistence gene is associated with dairy consumption opening the way to Mendelian randomization [85], [86]. If our findings prove to be robust in future replications, the public health implications could be profound with the promotion of milk and dairy products as part of a healthy diet.

### Conclusion

In conclusion, our study showed an inverse association between daily milk intake and obesity suggesting that dietary patterns characterized by high milk intake may lower the risk of obesity in adult Indians. These findings need further corroboration by longitudinal and clinical studies but may well have public health significance in the Indian population.

### Data Sharing

No additional data available.

## References

[1] Bansal D, Satija A, Khandpur N, Bowen L, Kinra S, et al.. (2010) Effects of migration on food consumption patterns in a sample of Indian factory workers and their families. Public Health Nutr. 1–8. 10.1017/S1368980010001254 20507672

[2] Reddy KS, Yusuf S (1998) Emerging epidemic of cardiovascular disease in developing countries. Circulation 97(6): 596–601. 9494031 10.1161/01.cir.97.6.596

[3] Yadav K, Krishnan A (2008) Changing patterns of diet, physical activity and obesity among urban, rural and slum populations in north India. Obes Rev 9(5): 400–8. 18627500 10.1111/j.1467-789X.2008.00505.x

[4] Srinath Reddy K, Shah B, Varghese C, Ramadoss A (2005) Responding to the threat of chronic diseases in India. The Lancet 366(9498): 1744–9. 10.1016/S0140-6736(05)67343-6 16291069

[5] World Health Organisation WHO Infobase. Available: https://apps.who.int/infobase/Index.aspx. Accessed 2011 Sept 22.

[6] World Health Organization (2010) WHO Global Infobase: Data For Saving Lives. Available: http://www.who.int/infobase/report. Accessed 2012 Oct 13.

[7] Haslam DW, James WP (2005) Obesity. Lancet 366(9492): 1197–209. 16198769 10.1016/S0140-6736(05)67483-1

[8] Pingali P, Khwaja Y (2004) Globalisation of Indian diets and the transformation of food supply systems. Inaugural Keynote Address on 17th Annual Conference of the Indian Society of Agricultural Marketing; 5–7 Feb; Hyderabad: India. ESA Working Paper No. 04–05, Agricultural and Development Economics Division, the Food and Agriculture Organization, the United Nations.

[9] World Health Organisation (2003) Diet, nutrition and the prevention of chronic diseases. Report of a joint WHO/FAO expert consultation. Geneva: WHO, 2003. Available: http://whqlibdoc.who.int/trs/WHO_TRS_916.pdf. Accessed 2011 April 16.

[10] Mirmiran P, Esmaillzadeh A, Azizi F (2005) Dairy consumption and body mass index: an inverse relationship. Int J Obes 29(1): 115–21. 10.1038/sj.ijo.0802838 15534616

[11] Elwood PC (2005) Time to value milk. Int J Epidemiol 34(5): 1160–2. 16027143 10.1093/ije/dyi136

[12] Newby PK, Muller D, Hallfrisch J, Qiao N, Andres R, et al (2003) Dietary patterns and changes in body mass index and waist circumference in adults. Am J Clin Nutr 77(6): 1417–25. 12791618 10.1093/ajcn/77.6.1417

[13] Olivares S, Kain J, Lera L, Pizarro F, Vio F, et al (2004) Nutritional status, food consumption and physical activity among Chilean school children: a descriptive study. Eur J Clin Nutr 58(9): 1278–85. 15054403 10.1038/sj.ejcn.1601962

[14] Barba G, Troiano E, Russo P, Venezia A, Siani A (2005) Inverse association between body mass and frequency of milk consumption in children. Br J Nutr 93(1): 15–9. 15705220 10.1079/bjn20041300

[15] Shi LL, Tarrant M, Hui LL, Kwok MK, Lam TH, et al (2011) The role of milk and dairy products in childhood obesity: evidence from the Hong Kong’s “children of 1997” birth cohort. J Epidemiol Community Health 65: A16.

[16] Zemel MB, Donnelly JE, Smith BK, Sullivan DK, Richards J, et al (2008) Effects of dairy intake on weight maintenance. Nutr Metab 5: 28. 10.1186/1743-7075-5-28 PMC2579293 18950508

[17] Azadbakht L, Esmaillzadeh A (2008) Dietary and non-dietary determinants of central adiposity among Tehrani women. Public Health Nutr 11(5): 528–34. 17764604 10.1017/S1368980007000882

[18] Harvey-Berino J, Gold BC, Lauber R, Starinski A (2005) The impact of calcium and dairy product consumption on weight loss. Obes Res 13(10): 1720–6. 16286519 10.1038/oby.2005.210

[19] Phillips SM, Bandini LG, Cyr H, Colclough-Douglas S, Naumova E, et al (2003) Dairy food consumption and body weight and fatness studied longitudinally over the adolescent period. Int J Obes Relat Metab Disord 27(9): 1106–13. 12917718 10.1038/sj.ijo.0802370

[20] Snijder MB, van der Heijden AA, van Dam RM, Stehouwer CDA, Hiddink GJ, et al (2007) Is higher dairy consumption associated with lower body weight and fewer metabolic disturbances? The Hoorn Study. Am J Clin Nutr 85: 989–95. 17413097 10.1093/ajcn/85.4.989

[21] Berkey CS, Rockett HRH, Willett WC, Colditz GA (2005) Milk, Dairy Fat, Dietary Calcium, and Weight Gain A Longitudinal Study of Adolescents. Arch Pediatr Adolesc Med 159: 543–550. 15939853 10.1001/archpedi.159.6.543

[22] Lahti-Koski M, Pietinen P, Heliövaara M, Vartiainen E (2002) Associations of body mass index and obesity with physical activity, food choices, alcohol intake, and smoking in the 1982–1997 FINRISK Studies. Am J Clin Nutr 75: 809–17. 11976153 10.1093/ajcn/75.5.809

[23] Alonso A, Beunza JJ, Delgado-Rodríguez M, Martínez JA, Martínez-González MA (2005) Low-fat dairy consumption and reduced risk of hypertension: the Seguimiento Universidad de Navarra (SUN) cohort. Am J Clin Nutr 82(5): 972–9. 16280427 10.1093/ajcn/82.5.972

[24] Department for Environment, Food and Rural Affairs (2007) Desktop Study into Demand for Dairy Products. Available: http://www.defra.gov.uk/foodfarm/food/industry/sectors/milk/pdf/ agraceasreport.pdf Accessed 2012 Jan 7.

[25] Food and Agricultural Organization. FAOSTAT, Food Balance Sheets. Available: http://faostat.fao.org/site/368/default.aspx#ancor. Accessed 2012 Jan 7.

[26] Wiley AS (2011) Milk for “growth”. : global and local meanings of milk consumption in China, India, and the U.S. Food and Foodways 19(1): 11–33.

[27] Hoppe C, Mølgaard C, Michaelsen KF (2006) Cow’s Milk and Linear Growth in industrialized and Developing Countries. Annual Review of Nutrition 26: 131–173 DOI:10.1146/annurev.nutr.26.010506.103757. 10.1146/annurev.nutr.26.010506.103757 16848703

[28] Wang Y, Li S (2008) Worldwide trends in dairy production and consumption and calcium intake: is promoting consumption of dairy products a sustainable solution for inadequate calcium intake? Food Nutr Bull 29(3): 172–85. 18947030 10.1177/156482650802900303

[29] National Sample Survey Organisation (2007) Nutritional Intake in India, 2004–2005. Ministry of Statistics & Programme Implementation, Government of India.

[30] Ministry of Statistics and Programme Implementation, Government of India (2012) Household consumption of various goods and services in India-National Sample Survey 66th Round. NSS Report no. 541 (66/1.0/3), National Sample Survey organisation, NSO, India.

[31] Speedy AW (2003) Global Production and Consumption of Animal Source Foods. J Nutr 133: 4048S–4053. 14672310 10.1093/jn/133.11.4048S

[32] Cecchini M, Sassi F, Lauer JA, Lee YY, Guajardo-Barron V, et al (2010) Tackling of unhealthy diets, physical inactivity, and obesity: health effects and cost-effectiveness. Lancet 376(9754): 1775–84. 21074255 10.1016/S0140-6736(10)61514-0

[33] Ebrahim S, Kinra S, Bowen L, Andersen E, Ben-Shlomo Y, et al (2010) The effect of rural-to-urban migration on obesity and diabetes in India: a cross-sectional study. PLoS Med 7: e1000268. 20436961 10.1371/journal.pmed.1000268 PMC2860494

[34] Kinra S, Bowen LJ, Lyngdoh T, Prabhakaran D, Reddy KS, et al (2010) Sociodemographic patterning of non-communicable disease risk factors in rural India: a cross sectional study. BMJ 341: c4974. 20876148 10.1136/bmj.c4974 PMC2946988

[35] Lyngdoh T, Kinra S, Shlomo YB, Reddy S, Prabhakaran D, et al (2006) Sib-recruitment for studying migration and its impact on obesity and diabetes. Emerg Themes Epidemiol 3: 2. 16533387 10.1186/1742-7622-3-2 PMC1468400

[36] Reddy KS, Prabhakaran D, Chaturvedi V, Jeemon P, Thankappan KR, et al (2006) Methods for establishing a surveillance system for cardiovascular diseases in Indian industrial populations. Bull World Health Organ 84: 461–469. 16799730 10.2471/blt.05.027037 PMC2627369

[37] Indian Consensus Group (1996) Indian consensus for prevention of hypertension and coronary heart disease. A joint scientific statement of Indian Society of Hypertension and International College of Nutrition. J Nutr Environ Med 6: 309–318.

[38] Misra A, Chowbey PK, Makkar BM, Vikram NK, Wasir JS, et al (2009) Consensus statement for diagnosis of obesity, abdominal obesity and the metabolic syndrome for Asian Indians and recommendations for physical activity, medical and surgical management. J Assoc Physicians India 57: 163–70. 19582986

[39] Kumar S, Khunti K, Hanif W, Zaman J (2009) Position statement on diagnosis and treatment of obesity in British South Asians. Available: http://www.sahf.org.uk/uploads/docs/newsdocs/10.doc.

[40] Bowen L, Ebrahim S, De Stavola B, Ness A, Kinra S, et al (2011) Dietary intake and rural-urban migration in India: a cross-sectional study. PLoS One 6(6): e14822. 21731604 10.1371/journal.pone.0014822 PMC3120774

[41] Bowen L, Ebrahim S, De Stavola B, Ness A, Kinra S, et al (2012) Development and evaluation of a semi-quantitative food frequency questionnaire for use in urban and rural India. Asia Pacific J Clin 2012 21(3): 355–60. 22705424

[42] Parr CL, Veierød MB, Laake P, Lund E, Hjartåker A (2006) Test-retest reproducibility of a food frequency questionnaire (FFQ) and estimated effects on disease risk in the Norwegian Women and Cancer Study (NOWAC). Nutr J 5: 4. 16448553 10.1186/1475-2891-5-4 PMC1434764

[43] Marchioni DML, Voci SM, de Lima FEL, Fisberg RM, Slater B (2007) Reproducibility of a food frequency questionnaire for adolescents. Cad Saude Publica 23: 2187–2196. 17700953 10.1590/s0102-311x2007000900026

[44] Sudha V, Radhika G, Sathya RM, Ganesan A, Mohan V (2006) Reproducibility and validity of an interviewer-administered semi-quantitative food frequency questionnaire to assess dietary intake of urban adults in southern India. Int J Food Sci Nutr 57: 481–493. 17162327 10.1080/09637480600969220

[45] Hebert JR, Gupta PC, Mehta H, Ebbeling DB, Bhonsle RR, et al.. (2000) Sources of variability in dietary intake in two distinct regions of rural India: implications for nutrition study design and interpretation. Eur J Clin Nutr 54, 479–486. 10.1038/sj.ejcn.1601042 10878649

[46] Subramanian SV, Davey Smith G, Subramanyam M (2006) Indigenous health and socioeconomic status in India. PLoS Med 3(10): e421. 17076556 10.1371/journal.pmed.0030421 PMC1621109

[47] Subramanian SV, Nandy S, Irving M, Gordon D, Lambert H, et al (2006) The mortality divide in India: the differential contributions of gender, caste, and standard of living across the life course. Am J Public Health 96: 818–825. 16571702 10.2105/AJPH.2004.060103 PMC1470604

[48] Bharathi AV, Sandhya N, Vaz M (2000) The development & characteristics of a physical activity questionnaire for epidemiological studies in urban middle class Indians. Indian J Med Res 111: 95–102. 10937385

[49] Bharathi AV, Kuriyan R, Kurpad AV, Thomas T, Ebrahim Shah, et al (2010) Assessment of physical activity using accelerometry, an activity diary, the heart rate method and the Indian migration study questionnaire in south Indian adults. Public Health Nutr 13(1): 47–53. 19656418 10.1017/S1368980009005850

[50] Carlin JB, Gurrin LC, Sterne JA, Morley R, Dwyer T (2005) Regression models for twin studies: a critical review. Int J Epidemiol 34: 1089–99. 16087687 10.1093/ije/dyi153

[51] Malik VS, Sun Qi, Van Dam RM, Rimm EB, Willet WC, et al (2011) Adolescent dairy product consumption and risk of type 2 diabetes in middle-aged women. Am J Clin Nutr 94(3): 854–61. 21753066 10.3945/ajcn.110.009621 PMC3155931

[52] Ensminger AH, Esminger MKJ (1986) Food for Health: A Nutrition Encyclopedia. Clovis, California: Pegus Press.

[53] Dougkas A, Reynolds CK, Givens ID, Elwood PC, Minihane AM (2011) Associations between dairy consumption and body weight: a review of the evidence and underlying mechanisms. Nutrition Research Reviews 24(1): 72–95. 21320381 10.1017/S095442241000034X

[54] Satija A, Taylor FC, Khurana S, Tripathy V, Khandpur N, et al (2012) Differences in consumption of food items between obese and normal-weight people in India. Natl Med J India 25: 10–13. 22680313

[55] Shaper AG, Wannamethee G, Walker M (1991) Milk, butter and heart disease. BMJ 302: 786–7. 10.1136/bmj.302.6779.785 PMC1669543 2021772

[56] Abbott RD, Curb JD, Rodriguez BL, Sharp DS, Burchfiel CM, et al (1996) Effect of dietary calcium and milk consumption on risk of thrombo-embolic stroke in older middle aged men. The Honolulu Heart Program. Stroke 27: 813–8. 8623098 10.1161/01.str.27.5.813

[57] Bostic RM, Kushi LH, Wu Y, Meyer KA, Sellers TA, et al (1999) Relation of calcium, vitamin D, and dairy food intake to ischaemic heart disease mortality among postmenopausal women. Am J Epidemiol 149: 151–61. 9921960 10.1093/oxfordjournals.aje.a009781

[58] Ness AR, Davey Smith G, Hart C (2001) Milk, coronary heart disease and mortality. J Epidemiol Community Health 55: 379–82. 11350992 10.1136/jech.55.6.379 PMC1731907

[59] Zemel MB, Shi H, Greer B, Dirienzo D, Zemel PC (2000) Regulation of adiposity by dietary calcium. FASEB J 14: 1132–38. 10834935

[60] Heaney RP, Davies KM, Barger-Lux MJ (2002) Calcium and weight: clinical studies. J Am Coll Nutr 21: 152S–5S. 11999544 10.1080/07315724.2002.10719213

[61] Tremblay A, Gilbert JA (2009) Milk products, insulin resistance syndrome and type 2 diabetes. J Am Coll Nutr 28(1): 91S–102S. 19571167 10.1080/07315724.2009.10719809

[62] Zemel MB, Thompson W, Milstead A, Morris K, Campbell P (2004) Dietary calcium and dairy products accelerate weight and fat loss during energy restriction in obese adults. Obes Res 12: 582–90. 15090625 10.1038/oby.2004.67

[63] Vernarelli JA, Lambert JD (2012) Tea consumption is inversely associated with weight status and other markers for metabolic syndrome in US adults. Eur J Nutr [Epub ahead of print]. 10.1007/s00394-012-0410-9 PMC3515715 22777108

[64] Kovacs EM, Lejeune MP, Nijs I, Westerterp-Plantenga MS (2004) Effects of green tea on weight maintenance after body-weight loss. Br J Nutr. 91(3): 431–7. 10.1079/BJN20041061 15005829

[65] Hursel R, Viechtbauer W, Westerterp-Plantenga MS (2009) The effects of green tea on weight loss and weight maintenance: a meta-analysis. Int J Obes (Lond). 33(9): 956–61. 10.1038/ijo.2009.135 19597519

[66] Louie JCY, Flood VM, Hector DJ, Rangan AM, Gill TP (2011) Dairy consumption and overweight and obesity: a systematic review of prospective cohort studies. Obesity Reviews 12(7): e582–e592. 21521450 10.1111/j.1467-789X.2011.00881.x

[67] Pereira MA, Jacobs DR Jr, Van Horn L, Slattery ML, Kartashov AI, et al (2002) Dairy consumption, obesity, and the insulin resistance syndrome in young adults: the CARDIA Study. JAMA 287: 2081–2089. 11966382 10.1001/jama.287.16.2081

[68] Rajpathak SN, Rimm EB, Rosner B, Willett WC, Hu FB (2006) Calcium and dairy intakes in relation to long-term weight gain in US men. Am J Clin Nutr 83: 559–566. 16522901 10.1093/ajcn.83.3.559

[69] Rosell M, Hakansson NN, Wolk A (2006) Association between dairy food consumption and weight change over 9 years in 19 352 perimenopausal women. Am J Clin Nutr 84: 1481–1488. 17158433 10.1093/ajcn/84.6.1481

[70] Snijder MB, van Dam RM, Stehouwer CD, Hiddink GJ, Heine RJ, et al (2008) A prospective study of dairy consumption in relation to changes in metabolic risk factors: the Hoorn Study. Obesity (Silver Spring) 16: 706–709. 18239556 10.1038/oby.2007.93

[71] Vergnaud A-C, Peneau S, Chat-Yung S, Kesse E, Czernichow S, et al (2008) Dairy consumption and 6-year changes in body weight and waist circumference in middle-aged French adults. Am J Clin Nutr 88: 1248–1255. 18996859 10.3945/ajcn.2007.25151

[72] Drapeau V, Despres JP, Bouchard C, Allard L, Fournier G, et al (2004) Modifications in food-group consumption are related to long-term body-weight changes. Am J Clin Nutr 80: 29–37. 15213024 10.1093/ajcn/80.1.29

[73] Willett WC (1998) Nutritional Epidemiology. New York: Oxford University Press.

[74] Hebert JR, Gupta PC, Bhonsle RB, Murti PR, Mehta H, et al (1998) Development and testing of a quantitative food frequency questionnaire for use in Kerala, India. Public Health Nutr 1(2): 123–130. 10933409 10.1079/phn19980019

[75] Hebert JR, Gupta PC, Bhonsle RB, Sinor PN, Mehta H, et al (1999) Development and testing of a quantitative food frequency questionnaire for use in Gujarat, India. Public Health Nutr 2(1): 39–50. 10452730 10.1017/s1368980099000051

[76] Pandey D, Bhatia V, Boddula R, Singh HK, Bhatia E (2005) Validation and reproducibility of a food frequency questionnaire to assess energy and fat intake in affluent north Indians. Natl Med J India 18: 230–235. 16433134

[77] Yajnik CS, Janipalli CS, Bhaskar S, Kulkarni SR, Freathy RM, et al (2009) FTO gene variants are strongly associated with type 2 diabetes in South Asian Indians. Diabetologia 52: 247–52. 19005641 10.1007/s00125-008-1186-6 PMC2658005

[78] Loos RJ, Lindgren CM, Li S, Wheeler E, Zhao JH, et al (2008) Common variants near MC4R are associated with fat mass, weight and risk of obesity. Nat Genet 40: 768–75. 18454148 10.1038/ng.140 PMC2669167

[79] Taylor AE, Sandeep MN, Janipalli CS, Giambartolomei C, Evans DM, et al.. (2011) Associations of FTO and MC4R Variants with Obesity Traits in Indians and the Role of Rural/Urban Environment as a Possible Effect Modifier. Journal of Obesity doi:10.1155/2011/307542 10.1155/2011/307542 PMC3139181 21785715

[80] International Institute for Population Sciences (IIPS) and Macro International (2007) National Family Health Survey (NFHS–3), 2005–06: Volume I. India. Mumbai: IIPS.

[81] Census of India (2011) Registrar General of India. Available: www.censusofindia.gov.in.

[82] Willett WC, Howe GR, Kushi LH (1997) Adjustment for total energy intake in epidemiologic studies. Am J Clin Nutr 65: 1220S–1231S. 9094926 10.1093/ajcn/65.4.1220S

[83] Davey Smith G, Ebrahim S (2004) Mendelian randomization: prospects, potentials, and limitations. Int J Epidemiol 33: 30–42. 15075143 10.1093/ije/dyh132

[84] Davey Smith G, Lawlor DA, Timpson NJ, Baban J, Kiessling M, et al (2009) Lactase persistence-related genetic variant: population substructure and health outcomes. Eur J Hum Genet 17(3): 357–67. 18797476 10.1038/ejhg.2008.156 PMC2986166

[85] Timpson NJ, Brennan P, Gaborieau V, Moore L, Zaridze D, et al (2010) Can lactase persistence genotype be used to reassess the relationship between renal cell carcinoma and milk drinking? Potentials and problems in the application of Mendelian randomization. Cancer Epidemiol Biomarkers Prev. 19(5): 1341–8. 10.1158/1055-9965.EPI-09-1019 PMC4141143 20447925

[86] Laaksonen MM, Mikkilä V, Räsänen L, Rontu R, Lehtimäki TJ, et al (2009) Cardiovascular Risk in Young Finns Study Group. Genetic lactase non-persistence, consumption of milk products and intakes of milk nutrients in Finns from childhood to young adulthood. Br J Nutr. 102(1): 8–17. 10.1017/S0007114508184677 19138442

